# Advancing measurement and monitoring of reproductive, maternal, newborn and child health and nutrition: global and country perspectives

**DOI:** 10.1136/bmjgh-2019-001512

**Published:** 2019-06-24

**Authors:** Tanya Marchant, Zulfiqar A Bhutta, Robert Black, John Grove, Catherine Kyobutungi, Stefan Peterson

**Affiliations:** 1 Department of Disease Control, London School of Hygiene & Tropical Medicine, London, UK; 2 Sick Kids, University of Toronto, Toronto, Ontario, Canada; 3 Aga Khan University, Karachi, Pakistan; 4 Institute for International Programs, Johns Hopkins Bloomberg School of Public Health, Baltimore, Maryland, USA; 5 World Health Organization, Geneva, Switzerland; 6 African Population and Health Research Centre, Nairobi, Kenya; 7 UNICEF, Health Section, Programme Division, New York City, New York, USA

**Keywords:** epidemiology, public health

## Introduction

Aligned with the Sustainable Development Goals, the Global Strategy for Women’s, Children’s and Adolescents’ Health (2016–2030) represents an essential shift in prioritisation for actions designed to help families live healthy, secure lives and fulfil their economic potential.^1^ The reproductive, maternal, newborn, child and adolescent health and nutrition (RMNCAH-N) agenda is now both broader and more complex than was the case during the Millennium Development Goal era, creating a need for new data. To contribute to this need, Countdown to 2030 for Women’s, Children’s and Adolescents’ Health (Countdown), a multi-institutional network of academics from institutions around the world and representatives from United Nations agencies and civil society, aims to enhance monitoring and measurement of women’s, children’s and adolescents’ health globally and in countries.^2^ In 2018, Countdown organised a measurement conference in Stellenbosch, South Africa, that brought together 100 experts in multiple areas of RMNCAH-N, which resulted in the six papers in this supplement and an overall research agenda.

The manuscripts in this collection represent the first developments of Countdown’s work to enhance measurement. They identify some of the persistent measurement and monitoring gaps in RMNCAH-N, for example, by reviewing the evidence on methods for generating effective coverage estimates and presenting actionable analytical methods to identify inequalities within and between countries. The collection also considers measurement advances for early childhood development and for nutrition. Further, it expands to analyse new priority issues, including using national surveys to analyse the impact of armed conflicts on RMNCAH-N;^3^ and describing the new data needed to better understand the social, political and contextual complexity of health system governance.

Countdown will continue to extend this measurement improvement agenda. In some aspects, however, the measurement and monitoring of RMNCAH-N is more advanced than other health areas, such as infectious diseases, non-communicable diseases, injuries and mental health. Many indicators of service contact and mortality are collected through surveys and can be disaggregated by multiple dimensions of inequality. Indeed, the inequality component of the Universal Health Coverage service coverage index is almost entirely based on RMNCAH-N indicators.^4^ Major gaps remain, however, in terms of service quality and effective coverage, maternal mortality, morbidity and causes of deaths, cognitive development and multiple other indicators of child well-being, and multisectoral service provision.

Beyond the technical detail of each field, the papers in the collection broadly share two common calls for measurement. First, the need for greater harmonisation of measurement standards, ideally underpinned by an authority such as WHO, as demonstrated by current endeavours in the field of maternal and newborn health, for example.^5^ Second, the need for investment in further development of measurement tools and methods. Both are plainly justified and align well with expert opinion.^6^ Consistent with Countdown’s commitment to situate more measurement work in countries and to help build domestic measurement expertise, harmonisation and investment have potential to advance agendas at both global and national levels. But, depending on perspective, there is the possibility of a tension between these two sets of needs.

## Harmonisation

A common theme across the manuscripts is the need for a process to generate global consensus on a minimum core set of validated coverage indicators on high-impact interventions, with guidance for measurement by WHO, and incorporated into relevant measurement tools. The case is well made that without this the interpretation and comparability of data across time and place would be limited, opportunities for learning reduced and potential for influence diminished. The review by Amouzou *et al* demonstrates an urgent need for harmonisation of definition and methods if we are to progress quality-adjusted coverage measurement from specialist studies to standard practice.^7^ For early childhood development, the need for a measurement framework and indicators to enable cross-country comparison of progress and help sustain momentum is clearly made.^8^ And with only half of high-impact nutrition interventions being measured through large-scale surveys, it is evident that programmes addressing malnutrition need more and standardised data.^9^

Gillespie *et al* also make the important point about the possible tension between harmonisation of indicators for global measurement and the indicator definition that speaks to a specific country programme. When measurement is driven by country priorities, the ideal indicators for programme management will depend on the intended use of the data, on the level and frequency of measurement, and on the desire to track progress over time by aligning with past measures. Within countries, governments need to be able to track their own progress and so need a consistent approach to measurement within their own setting. Flexibility in coding and indicator definition is needed to ensure that data can be analysed to meet both global and country needs. This issue is currently prominent for antenatal care as WHO has increased the recommended number of pregnancy contacts from four to eight antenatal visits,^10^ but most countries are yet to action such a transition and will continue to need to track coverage of at least four visits for some time to come. Similarly, the global definition and measurement of skilled attendance at birth is becoming more precise as quality-of-care issues are more prominent; but, in the face of acute human resource shortages and task-shifting policies, there continues to be considerable variability between country level definitions of the cadres considered to provide skilled care.^11^

## Investment

Across multiple topics, investment is needed for better, validated indicators that are integrated in standardised data collection methods with sufficiently large sample sizes for multiple disaggregation, while strengthening country capacity in data analysis and use, to ultimately aid data-informed decision-making and implementation. Whether implicit or explicit, the language of this call for investment primarily focused on investment in better periodic survey data rather than routine health information or indeed qualitative data sources.

For example, the agenda to increase the rate of progress in health by making sure that no one is left behind means that we need to be able to gain greater insight from data. This inevitably means larger household survey datasets with bigger sample sizes for more granular, disaggregated analysis. The analysis by Victora *et al* makes clear the added value of extending relative equity analysis from quintiles to deciles of households, or of examining intersectionality between categories of inequality, for example place of residence and socioeconomic status.^12^ This is important not least because of the positive evidence that slowly but surely inequities within and between countries are reducing—so that differences are becoming more subtle, more complex.

In addition to gaining greater use from surveys, there is also an imperative to invest in the country health information systems. Acknowledged as having potential to contribute to data for decision-making, data from these sources are frequently dismissed because of well-justified concerns about data quality or because of the constraint of working with imperfect denominators. Nonetheless, there are essential reasons for both global and national actors to look for investment to improve on this. First, most country programmes want to base decision-making on their own data and are motivated to build capacity to manipulate their own data; this is well aligned with global actor ambitions to support more effective country-led data-driven decision-making for health. Second, routine data can be available in real time and analysed at macro, meso or micro levels of granularity depending on needs and therefore uniquely suitable for real-time monitoring and course correction,^13^ again providing alignment for the global community to promote and support implementation science to increase the rate of progress in health. And third, there are many things that surveys cannot reliably measure because the respondents do not know the answer to questions, for example, treatment for illness or measures of healthcare quality.^14^ For measurement of clinical care of this sort, facility data sources need to be strengthened. And this would be to the benefit of the global community’s need for data that can be analysed to better estimate the potential of health gain that can be derived from contacts with the health service.^15^ The Countdown to 2030 has shifted its focus on collaborating with country public health institutions and ministries of health to generate evidence and strengthen analytical capacity through regional initiatives. The goal is to further expand these collaborations and strengthen the links with countries’ own reviews of progress and performance of the strategies and plans for women’s, children’s and adolescents’ health.

And finally, unpacking the drivers of health, as described by George *et al,* encourages reflection on the current framing of health and the information we use to inform our vision.^16^ For this, we need harmonised quantitative data that speak to a service delivery lens—be it survey or administrative—but also other types of data that speak to societal and systems lenses (eg, contextual data on organisational structures, social norms and the interdependence of actors). This agenda, defined and committed to by the Countdown community of measurement experts, needs new data and new combinations of disciplines working together, at global and national levels, to also capture and incorporate country-derived tacit knowledge.
